# Addressing the Burden and Management Strategies for Disparities and Inequities Among Liver Transplant Professionals: The ILTS Experience

**DOI:** 10.3389/ti.2023.11240

**Published:** 2023-06-02

**Authors:** Oya Andacoglu, Manhal Izzy, Dieter Adelmann, Victoria Aguilera, Chiara Becchetti, Marina Berenguer, Gabriella A. Berlakovich, Simantika Ghosh, Emmanouil Giorgakis, Nyingi Kemmer, Keri E. Lunsford, Iman F. Montasser, Martin I. Montenovo, Anna Mrzljak, Sher-Lu Pai, Irene Scalera, Nazia Selzner

**Affiliations:** ^1^ Division of Transplantation, Department of Surgery, The University of Oklahoma College of Medicine, University of Oklahoma, Oklahoma City, OK, United States; ^2^ Division of Gastroenterology, Hepatology, and Nutrition, Vanderbilt University Medical Center, Nashville, TN, United States; ^3^ Department of Anesthesia and Perioperative Care, UCSF School of Medicine, University of California, San Francisco, San Francisco, CA, United States; ^4^ Hepatology and Liver Transplant Unit, IIS La Fe and CIBER-EHD, University of Valencia, Universitary and Politecnic Hospital La Fe, Valencia, Spain; ^5^ University Clinic for Visceral Surgery and Medicine, Inselspital, University Hospital of Bern, Bern, Switzerland; ^6^ Division of Transplantation, Medical University of Vienna, Vienna, Austria; ^7^ Department of Anesthesiology, Narayana Health, Narayana, India; ^8^ Department of Surgery, University of Arkansas for Medical Sciences, Little Rock, AR, United States; ^9^ Division of Gastroenterology, Tampa General Hospital, Tampa, FL, United States; ^10^ Department of Surgery, Division of Transplant and HPB Surgery, Rutgers New Jersey Medical School, Newark, NJ, United States; ^11^ Department of Tropical Medicine, Ain Shams University, Cairo, Egypt; ^12^ Division of Hepatobiliary Surgery and Liver Transplant, Vanderbilt University Medical Center, Nashville, TN, United States; ^13^ Department of Gastroenterology and Hepatology, University Hospital Center Zagreb, Zagreb, Croatia; ^14^ Department of Anesthesiology and Perioperative Medicine, Mayo Clinic, Jacksonville, FL, United States; ^15^ Division of Hepatobiliary Surgery and Liver Transplant, University Hospital Policlinic of Bari, Bari, Italy; ^16^ Ajmera Transplant Center, University of Toronto, Toronto, ON, Canada

**Keywords:** liver transplantation, equity, diversity, inclusion, professional societies

## Abstract

Medical professional environments are becoming increasingly multicultural, international, and diverse in terms of its specialists. Many transplant professionals face challenges related to gender, sexual orientation or racial background in their work environment or experience inequities involving access to leadership positions, professional promotion, and compensation. These circumstances not infrequently become a major source of work-related stress and burnout for these disadvantaged, under-represented transplant professionals. In this review, we aim to 1) discuss the current perceptions regarding disparities among liver transplant providers 2) outline the burden and impact of disparities and inequities in the liver transplant workforce 3) propose potential solutions and role of professional societies to mitigate inequities and maximize inclusion within the transplant community.

## Introduction

Over the last few years, medical professional environments have seen a change with increasing workforce diversity due to immigration as well as exponential growth of women and minority populations among medical trainees. The positive impact of diversity is well recognized, and it is promising to see the evolving knowledge and research in this area (1–28)^1^; yet, many professionals continue to face discriminations related to gender, racial background, sexual orientation, or inequities in terms of access to leadership positions, compensation, or professional promotions. Active plans are thus needed by transplant stakeholders both at global and institutional scales to reduce discrimination and to promote female and minorities access to leadership positions. The current review focuses on the burden and impact of disparities and proposes potential solutions to mitigate these inequities.

## Current Perceptions About Equity, Diversity, and Inclusion Among Liver Transplantation Professionals and the Impact of Disparity and Inequity Among Liver Transplantation Workforce

**FIGURE 1:**
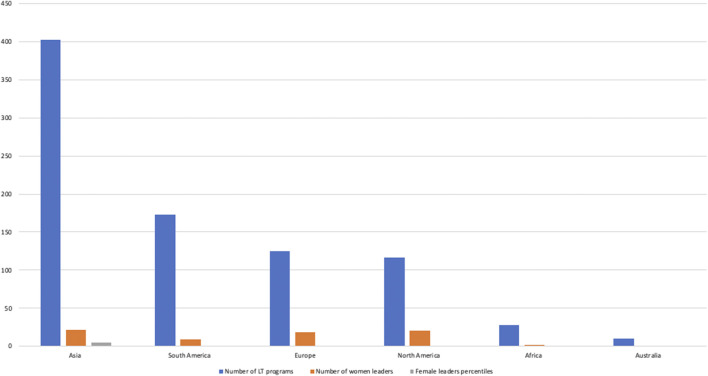
Global distribution of woman leadership in liver transplantation—adapted from Aguilera V et al. (1).

According to a recent survey by the Equity, Diversity, and Inclusion (EDI) Committee of the International Liver Transplantation Society (ILTS), 35% of liver transplantation (LT) professionals reported a form of discrimination (1). The reasons for very low rates of woman leadership are consistent across the reports (2–5). A survey by the ILTS EDI Committee, which included 243 transplant centers globally, reported that only 32 (13.2%) had at least 1 woman as the director of LT, chief of transplant surgery, or chief of transplant hepatology (6) while another survey found that woman leadership was present in only 8% of 856 transplant programs globally (1) (Figure 1). Lowest woman leadership was in transplant surgery followed by hepatology and anesthesia (14.2% vs. 20% vs. 32.1% respectively, *p* = 0.046) (1). Disparities are also notable in the academic sphere, affecting the proportion of female professionals and minorities represented in senior authorship and transplant journals’ editorial boards (7, 8).

Female surgeons face obstacles, not only in leadership but also advancing their technical skills; as surgeons they are more commonly assigned as assistant surgeon as opposed to being primary surgeon for complex cases; consequently, they end-up having overall less case numbers within the same specialty or less cases for complex cases (9–11). They also face obstacles based on societal perceptions and not having supportive systems for an equitable career growth (9–11). In relation to these issues, the high incidence of attrition experienced by early-career abdominal transplant surgeons is concerning (12). Similarly, while there is wide variety in hepatology workflow and compensation, a burn out percent of 35% among trainees pursuing careers in transplant hepatology is alarming (13).

### Effect of Race and Country of Origin on a Career in Liver Transplant

There is evidence to suggest that African American and Hispanic individuals are underrepresented in the field of medicine compared to their representation in the general population (14–16)^1^. For example, in the United States (US), African American and Hispanic individuals make up approximately 31% of the overall US population. Yet, the American Medical Association (AMA) reported in 2020, that approximately only 7% of all active physicians in the US identified as African American, and approximately 8% identified as Hispanic or Latino (14–16)^1^. On the other hand, among the surgical directors of LT programs, only 16% were Hispanic or African American (4). This disparity can be due to a range of factors, including social and economic disadvantages, lack of equitable access to educational and training opportunities compared to other groups, and discrimination. It is also possible that a person’s country of origin could impact their potential career in LT, depending on the availability of educational and training programs in that country and the existence of LT in the healthcare system. However, recent initiatives by multiple transplant societies to recognize transplant training of international graduates and offer them equal opportunity for training and certification processes would help to mitigate the disparities among international trainees. The impact of complete or relative lack of EDI in the field of LT can be quite consequential in hindering career development, limiting creativity and innovation among providers affected by this unfortunate reality. Therefore, it is important to recognize and address these issues to promote diversity and inclusion in the field of LT and ensure that individuals from all backgrounds have equal opportunities to enter and excel in this field for the betterment of LT as a specialty, us as a scientific community and importantly our patients.

## Highlights of the ILTS Experience

**FIGURE 2:**
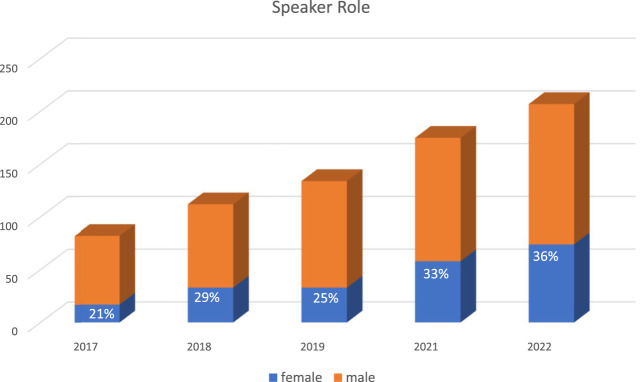
Gender Distribution of ILTS congresses speaker roles.

**FIGURE 3:**
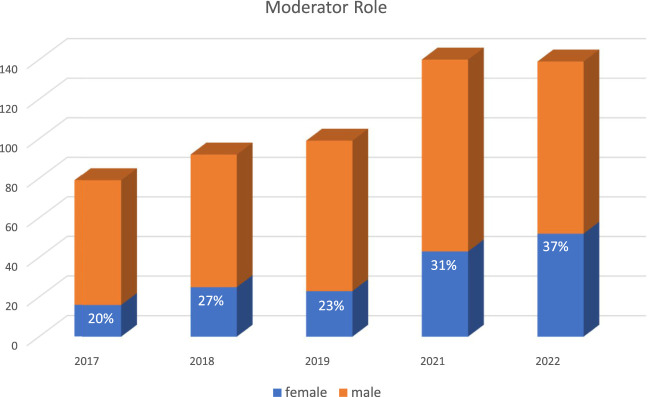
Gender Distribution of ILTS congresses moderator roles.

**FIGURE 4:**
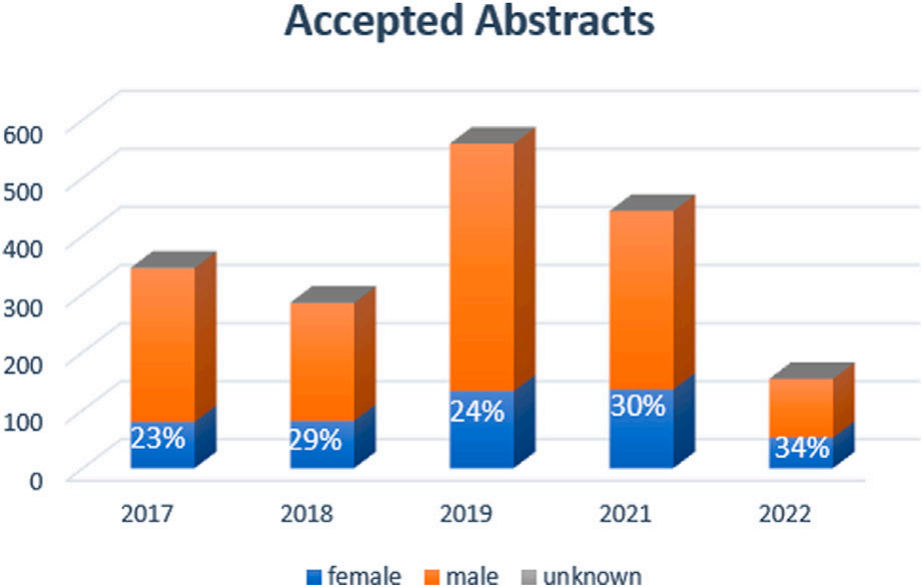
Gender Distribution of ILTS congresses accepted abstracts.

In reviewing the ILTS data, over the last 5 years (2017–2022), the female participation rate was 25%–31%, highest years being 2021 and 2022 at 31%. For the 2022 conference, although 21% did not specify their field, female participation was 45% in hepatology, 44% anesthesia and critical care followed by 20% rate in surgery. With the leadership and society efforts, female moderator and female speaker rates went up gradually and annually from 20% to 37% and 21% and 36%, respectively, between 2017–2022 (Figures 2, 3). Similarly, gender distribution of accepted abstracts reached 30% in the last year (Figure 4). Given 30% of total female attendance to the entire conference, speaker and moderator rates of 30% is a clear demonstration of the equitable representation of female participants in ILTS meetings which should be taken as a role model for all professional societies. Regarding country representation, six countries (USA, China, Republic of Korea, Turkey, United Kingdom, and India) accounted for between 50% and 57% of the total attendance in the previous 5 years (2017–2022), which may be reflecting limited access from underdeveloped countries to international medical conferences. Obviously, size of the country and total number of transplant programs would directly impact the participation to meetings. Regardless, attendance from Africa or the rest of the Asian continent remained low relative to North America and Europe. This is one of the areas ILTS and EDI committee is currently working on. For instance, in 2020, ILTS conducted an educational outreach initiative to help develop educational activities focused on the needs of specific regions around the world. The initiative is called the ILTS Regional Expansion of Advanced Learning (REAL) project. The aim of this initiative is to reach out to the different regions, mainly underdeveloped areas, where LT education is much needed, *via* educational programs tailored to what the key opinion leaders expressed (in prior surveys) as topics or areas of needed learning in their regions. REAL Asia was launched in 2020, REAL Latin America in 2021 and more recently REAL Africa 2022; representing a good example of the collaboration between the educational committee and EDI committee to maximize inclusiveness in educational initiatives.

Representation of women participants can be augmented both by encouraging more female professionals to participate in scientific events as well as encouraging the professional societies to be more inclusive of women in organizing committees and nominating them for moderator or chairperson roles and encouraging male professionals to recognize the achievements made by women in this challenging field. It is not a question of gender but a question of having the same unbiased opportunities within the given field.

## Strategies to Include Young Professionals in Transplant Societies

With increased representation of younger members in the field (17), they seem to be integrating into medical societies by creating subgroups or specific subcommittees in the various specialties. This can provide a good opportunity for younger members to establish their footprint as well as gain leadership skills. This would also serve to rejuvenate professional societies with new creative ideas. On the other hand, there needs to have mentors at the society level who can understand the expectations and needs of these of young members (18). ILTS founded the Young Investigators (YIs) subcommittee bringing together a variety of specialties that have expertise and training background in LT.

Online surveys have proven to be a tool used and appreciated by younger age groups (19). Despite several limitations in health epidemiology, they remain a valuable instrument for exploring trends (20). The role of online resources has become prominent in recent years and YIs rely upon online clinical resources in their practice (21). Even the use of social media has become very popular in the field of liver disease (22). In this respect, ILTS offers a wide range of online resources for its affiliates, stratified by macro-areas of interest (anesthesia, surgery, hepatology). Specifically, ILTS has promoted the Vanguard Committee. This committee’s mission is to promote the participation of younger members of the LT community in all ILTS activities, and to guide the society in responding to their educational and professional needs. Scopes of the committee include: to organize the part of the annual congress dedicated to YIs; to select the best published clinical and basic science research papers during the calendar year for the Vanguard Awards; to contribute to the Scientific Content in the monthly ILTS Newsletter; to assist in social media profile management and to lead monthly ILTS Vanguard Webinars on complex cases in LT.

### Solutions to Mitigate Inequities and Maximize Inclusion Within the Transplant Community

Multiple medical professional societies have initiated programs to improve diversity, equity, and inclusiveness for physicians and allied health professionals in recent years. These ongoing efforts have been developed as disparities ranging from work compensation (23, 24), manuscript publication conference speaker representation, academic promotion, to leadership position have all been identified in the field of medicine (25–28). The heightened awareness on racial and gender disparities has urged professional societies to be the physicians’ voice and organize the physicians to unite in the front of combating discriminations. In the field of LT, ILTS is no exception on being a driving force to fight against disparities and inequities.

LT is a unique medical field that integrates various medical specialties, and ILTS serves as a unifying entity that encourages collaborations between these physicians from different countries all over the world. In 2017, the ILTS created the Equity, Diversity, and Inclusion Committee to promote equity, diversity, and inclusion in LT. Since 2017, the committee has been utilizing the DMAIC (Define, Measure, Analyze, Improve, Control) (28) approach of Six Sigma to overcome the complex tasks of finding impactful EDI initiatives to reduce disparities among women and other racial or gender minorities.

When issues were raised after gathering feedback from the ILTS members, the EDI Committee defined the opportunities for improvement. Once the focus of each EDI project was identified, granular data were gathered to provide measurements of the existing issue. Subsequently, the data were analyzed to understand the scope of the problem and to determine the root causes of the issue. From each EDI project, the data gave ILTS the insight on how to reduce disparities and improve diversity. Whenever an initiative was put forward, ILTS EDI Committee continued to monitor progress after each project implementation to ensure success and look for other routes to further increase project impact. The data obtained from the EDI committee initiatives resulted in series of recommendations aiming to mitigate gender and racial disparities in LT practices (Table 1).

**TABLE 1:** Proposed international society initiatives to decrease disparities.

1. Acknowledge the existence of disparities at multiple levels
2. Prioritize society membership recruitment towards underrepresented groups
3. Modify the open-ended text field for members to accurately define their gender and ethnicity/race identity
4. Ensure adequate representation of gender and racial minorities for society participation including committee appointments, leadership positions, conference session speakers
5. Initiate mentorship programs, with focuses on trainees and junior physicians
6. Continue to increase awareness on EDI topics at the international level and collaborate with local EDI committees
7. Study disparities physician compensation
8. Promote adequate parental leave policies
9. Publish gathered data in international journals to increase visibility of EDI topics with recommendations on how to incorporate changes at local, national, and international levels
10. Journals to promote more women and under-represented groups to be included in editorial boards

## Conclusion

Significant disparities exist in the field of LT at multiple levels from leadership to training to societal representation. These disparities can have a remarkable impact on career development of the affected LT professionals. As ILTS and other international societies continue to provide initiatives, the support should be extended to local institutions aiming to mitigate inequities, strengthen the networking among underrepresented providers, and enhance optimal clinical practice, academic promotion, and leadership development in the field of LT.

## Footnotes

1 US Census data. *Explore Census Data: Learn about America’s People, Places, and Economy* (2020). Available from: https://data.census.gov/ (Accessed December 15, 2022).

## Abbreviations

ILTS, International Liver Transplant Society; EDI, Equity, Diversity, and Inclusion; LT, Liver Transplantation; USA, United States of America; YIs, Young Investigators.
